# Peak BMP Responses in the *Drosophila* Embryo Are Dependent on the Activation of Integrin Signaling

**DOI:** 10.1016/j.celrep.2015.10.079

**Published:** 2015-11-17

**Authors:** Annick Sawala, Margherita Scarcia, Catherine Sutcliffe, Scott G. Wilcockson, Hilary L. Ashe

(Cell Reports *12*, 1584–1593; September 8, 2015)

In the original version of this article, Figure 4E was inadvertently published with the incorrect Flag-TkvQD control western blot panel as a result of an error during figure preparation. The error does not alter the data interpretation. An error in the placement of the labels for Myc-EV in Figure 4J has also been corrected. The plus and minus signs were swapped in lanes 1 and 3. The corrected Figure 4 appears here and is now correct online as well. The authors apologize for any confusion caused by these errors.

**Figure 4:**
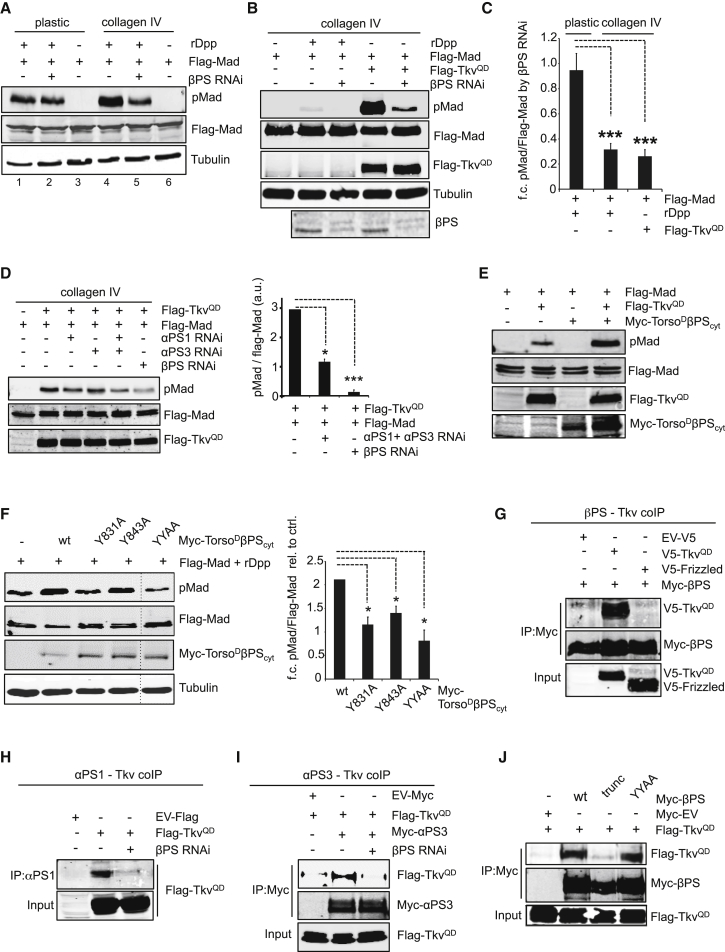
Molecular Mechanism of Integrin-Signaling-Mediated Enhancement of the BMP Pathway

